# Supplementary material for: The multi-functional reovirus σ3 protein is a virulence factor that suppresses stress granule formation and is associated with myocardial injury
Source: PLoS Pathog. 2021 Jul 8;17(7):e1009494. doi: 10.1371/journal.ppat.1009494 (PMC8291629; doi:10.1371/journal.ppat.1009494)
Supplement: S1 Table — (DOCX) [file ppat.1009494.s001.docx]

**Table S1** Commercial antibodies used in this study

| **Antibody** | **Type** | **Supplier** | **Cat.No.** |
| --- | --- | --- | --- |
| PKR (human) | Rabbit | Cell Signaling Technology | 3072S |
| PKR (mouse) | Mouse | Santa Cruz | sc-6282 |
| eIF2a | Rabbit | Santa Cruz | sc-11386 |
| PKRpT446 (mouse) | Rabbit | Upstate Cell Signaling | 07-532 |
| PKRpT446 (human) | Rabbit | Abcam | 32036 |
| eIF2apS51 | Rabbit | CST | 9721 |
| actin | Mouse | CST | 3700 |
| Flag | Mouse | Sigma-Aldrich | F3165 |
| HA | Mouse | Covance | MMS-101P |
| Myc | Mouse | BioLegend | 658502 |
| G3BP | Mouse | Abcam | 56574 |
| TIAR | Rabbit | CST | 5137 |
| TIA1 | Rabbit | Proteintech | 12133-2 |
| RNase L | Rabbit | Abcam | 191392 |
| Puromycin | Mouse | DSHB | PMY-2A4 |
