# Supplementary figures and images for: The multi-functional reovirus σ3 protein is a virulence factor that suppresses stress granule formation and is associated with myocardial injury
Source: PLoS Pathog. 2021 Jul 8;17(7):e1009494. doi: 10.1371/journal.ppat.1009494 (PMC8291629; doi:10.1371/journal.ppat.1009494)

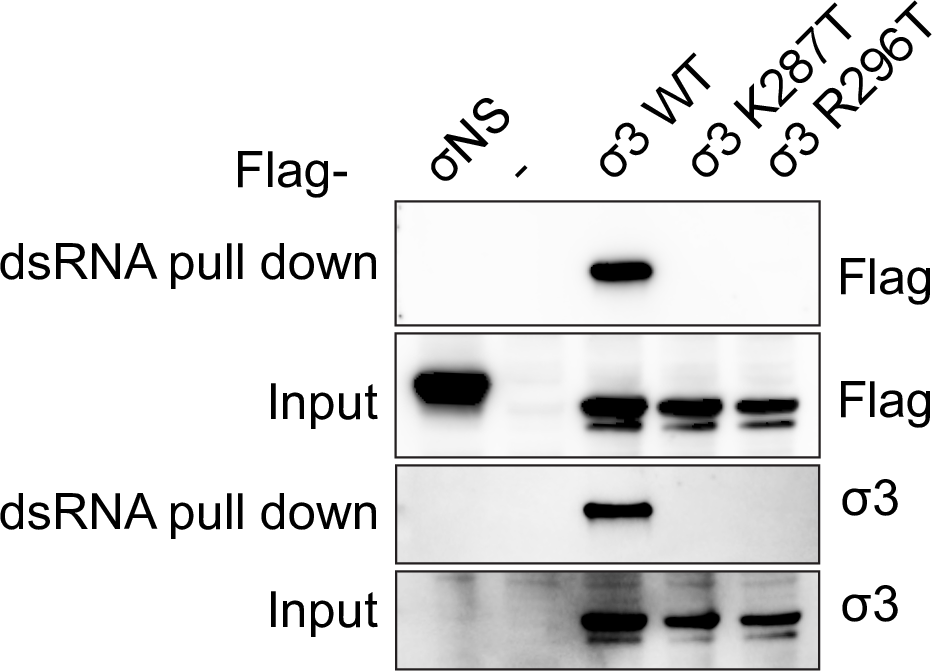

Supplement: S1 Fig — Biotinylated viral dsRNA extracted from purified T1L virions was incubated with cell lysates expressing Flag-tagged σNS or σ3 WT or mutant proteins. Streptavidin-agarose pull-downs were washed extensively and immunoblots were performed to detect input expression levels and the viral dsRNA-binding capacity of the indicated proteins. (TIF) [file ppat.1009494.s005.tif]

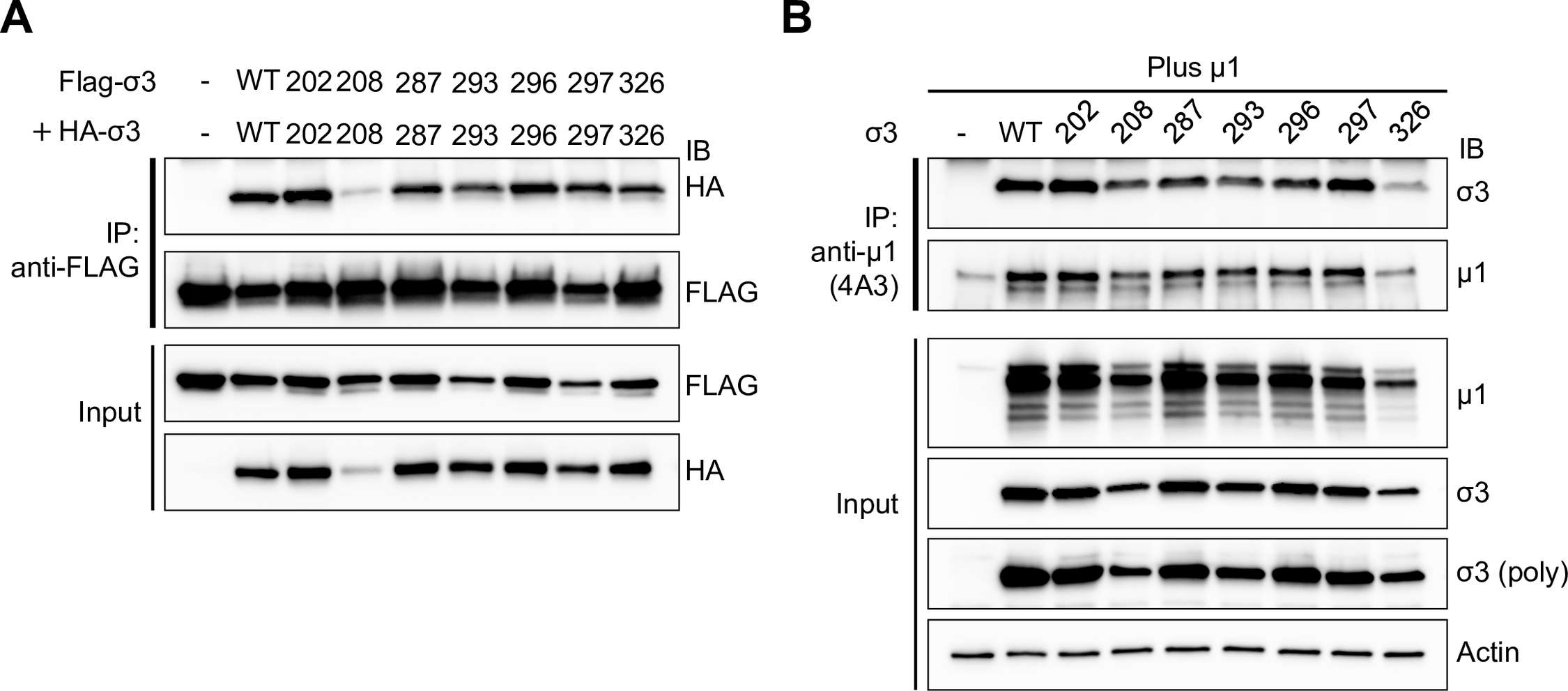

Supplement: S2 Fig — (A) dsRNA-binding defective mutants of σ3 remain capable of dimerizing. HA-tagged σ3 was co-transfected together with Flag-tagged σ3 into HEK293 cells. At 48 h pi, coimmunoprecipitation of HA-tagged σ3 with FLAG-tagged σ3 WT or dsRNA-binding defective mutants from cell lysates was performed using anti-Flag agarose beads for 2 h, followed by western blotting to detect indicated proteins. (B) dsRNA-binding defective mutants of σ3 are capable of assembling with co-expressed μ1. μ1 was transfected into HEK293 cells together with either σ3 WT or dsRNA-binding defective mutants. At 48 h post-transfection, coimmunoprecipitation of WT and dsRNA-binding mutants of σ3 was performed using a monoclonal antibody against μ1(4A3). Unless specified, σ3 was detected by monoclonal antibody (4F2). (TIF) [file ppat.1009494.s006.tif]

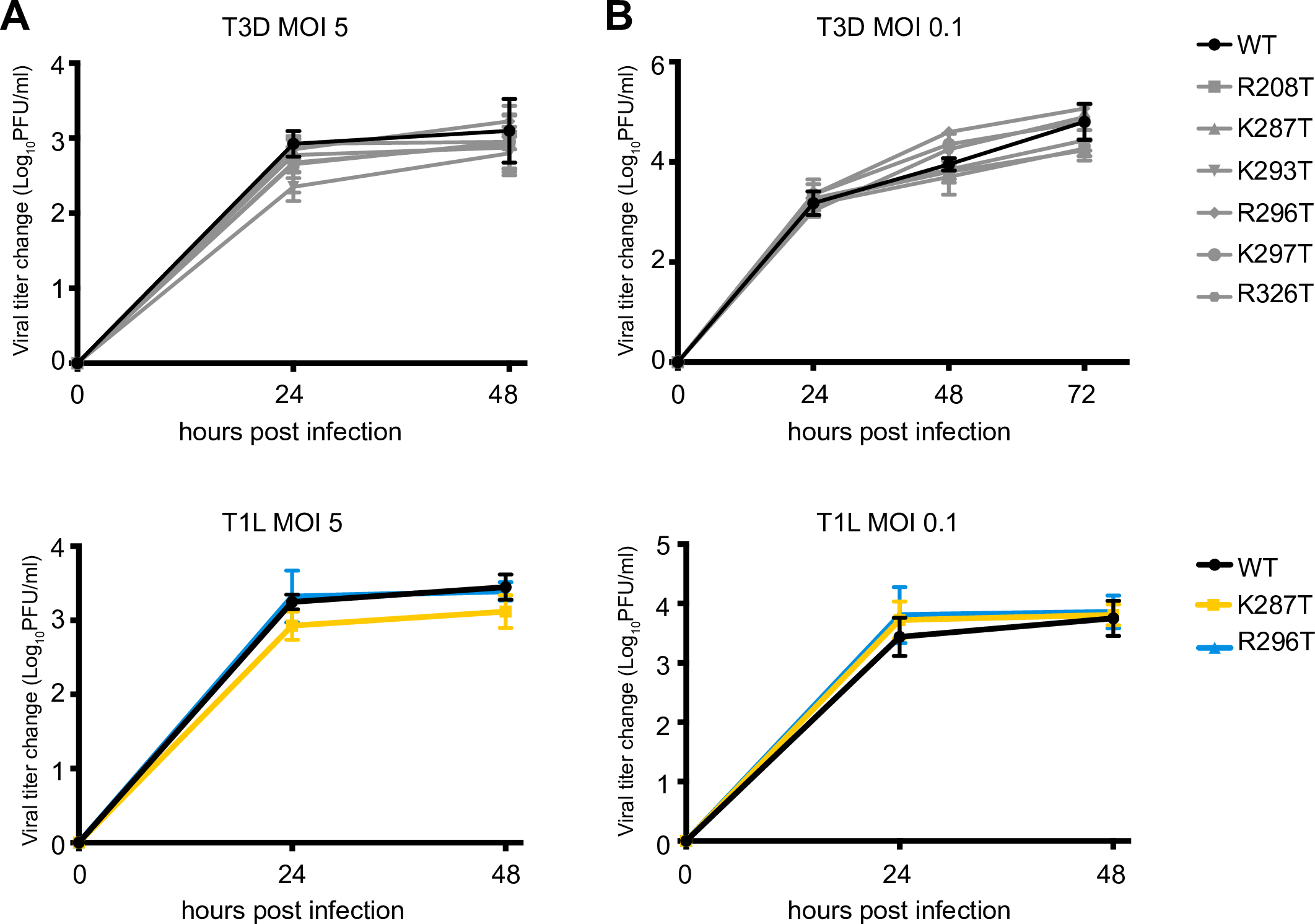

Supplement: S3 Fig — Recombinant T3D and T1L viruses that carry dsRNA-binding defective σ3 mutations grow with similar kinetics to WT virus during (A) single and (B) multiple step replication in L929 cells. Cells were infected with the indicated WT or mutant viruses at 5 PFU per cell (single step replication) or 0.1 PFU per cell (multiple step replication). Change in viral titer was determined by plaque assay. Data are reported as mean ± S.D. of three independent experiments. (TIF) [file ppat.1009494.s007.tif]

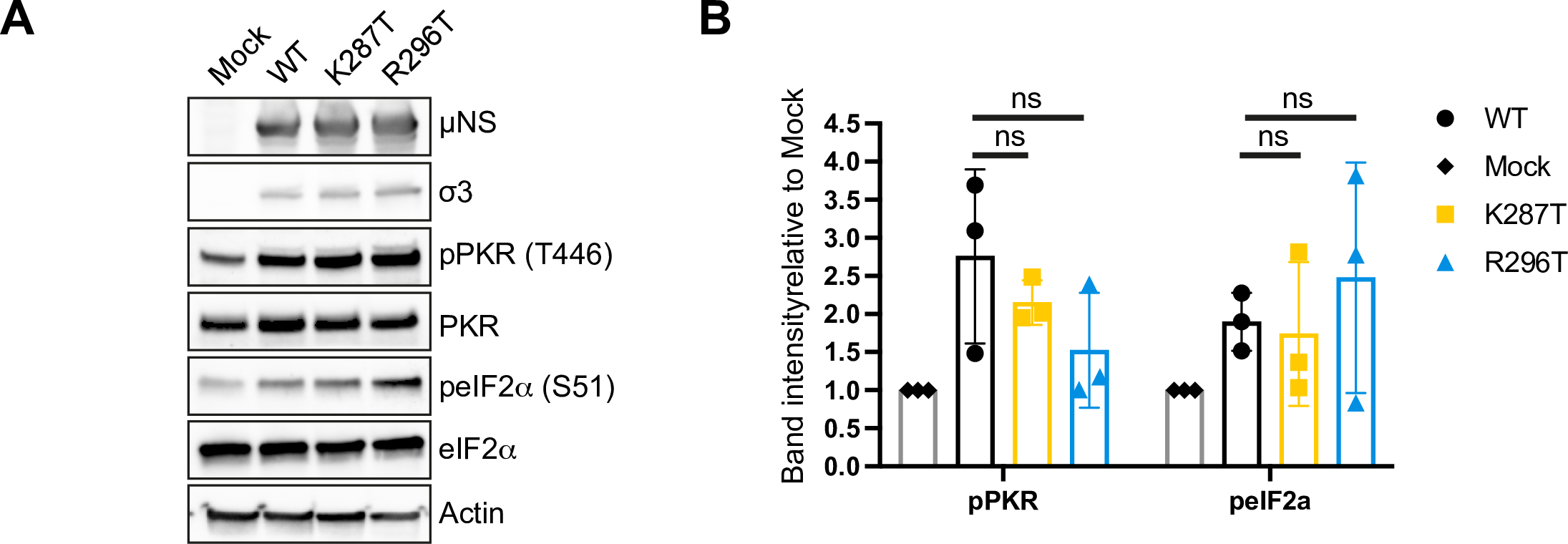

Supplement: S4 Fig — (A) Cells were mock-infected or infected with the indicated viruses at 5 PFU per cell. At 18 h pi, cell lysates were collected in denaturing lysis buffer for immunoblotting and probed with antibodies against viral proteins μNS and σ3, and cellular proteins PKR, phosphorylated PKR (T446), phosphorylated eIF2α (S51), and actin. (B) Band intensities of phosphorylated PKR and phosphorylated eIF2α were quantified and normalized to total PKR and eIF2α respectively, and then normalized to mock (mock = 1). Data shown represent the mean ± s.d. of three independent experiments. Multiple comparison, non-paired t tests were used to analyze differences compared to WT (ns = not significant). (TIF) [file ppat.1009494.s008.tif]

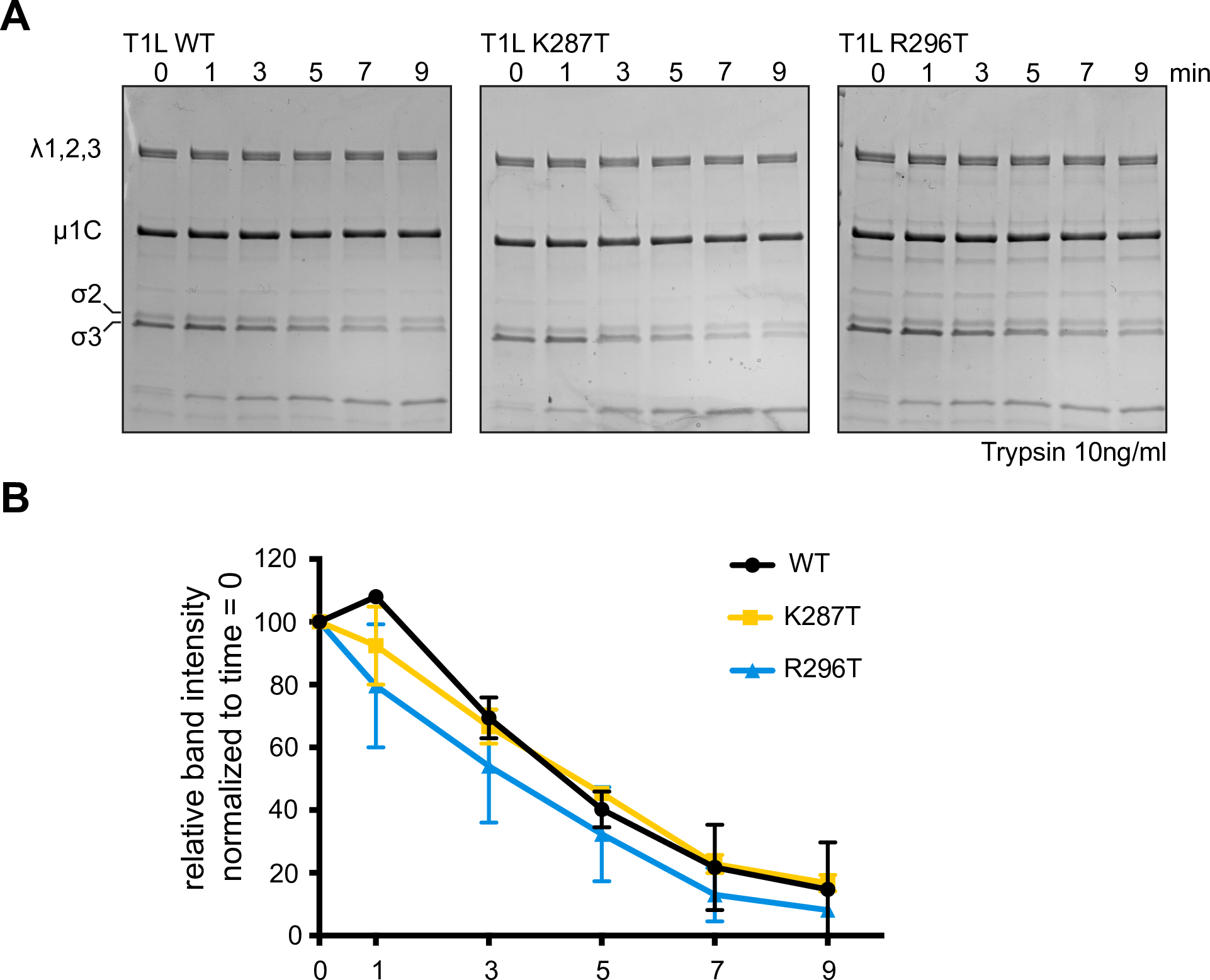

Supplement: S5 Fig — (A) Equal concentrations of purified virions were incubated with 10 ng/ml trypsin at 8°C. At the indicated time points, equivalent volumes of digestion aliquots were collected and analyzed by SDS-PAGE and Coomassie Brilliant Blue (representative experiment). (B) Band intensities of σ3 were measured using Image J software and normalized to the intensity at T = 0. Data shown represent the mean ± s.d. of two independent experiments. (TIF) [file ppat.1009494.s009.tif]

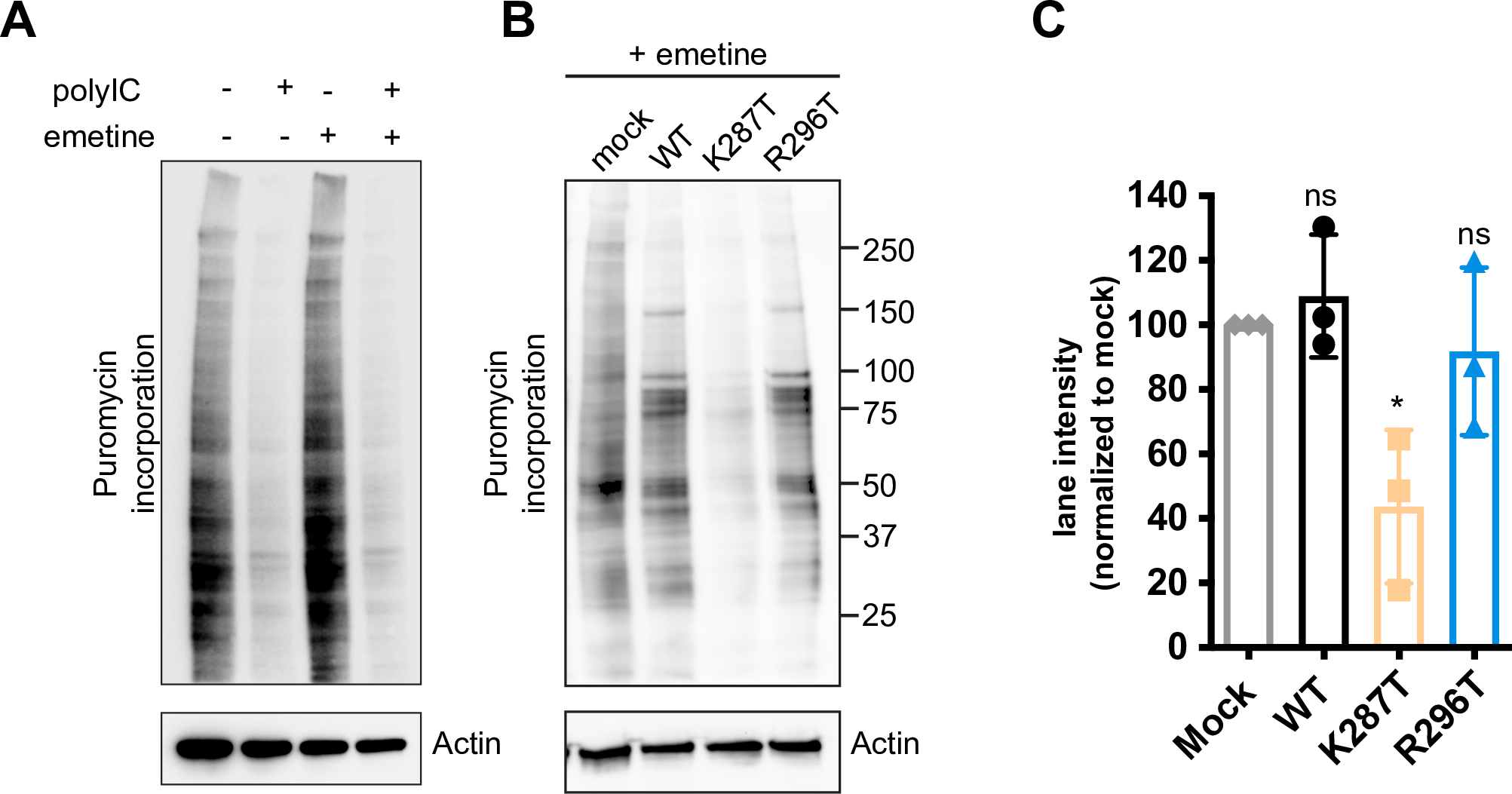

Supplement: S6 Fig — (A) A549 cells were left untreated or treated with poly (I:C) at 1 μg/ml for 6 h prior to treatment with 208 μM emetine for 15 min at 37°C followed by RPM labeling. Puromycin (PMY) incorporation levels were assessed by immunoblotting. (B) A549 cells were infected with T1L-WT, or T1L-K287T, or T1L-R296T at 100 PFU per cell. At 18 h pi, cells were treated with emetine for 15 min at 37°C followed by RPM labeling. PMY incorporation were assessed by immunoblotting. (C) Lane intensities were measured using Image J software and normalized to mock (mock was set to 100). Data shown represent the mean ± s.d. of three independent experiments. Multiple comparison, non-paired t tests were used to analyze differences comparing with mock. (ns = not significant; *, P < 0.05). (TIF) [file ppat.1009494.s010.tif]

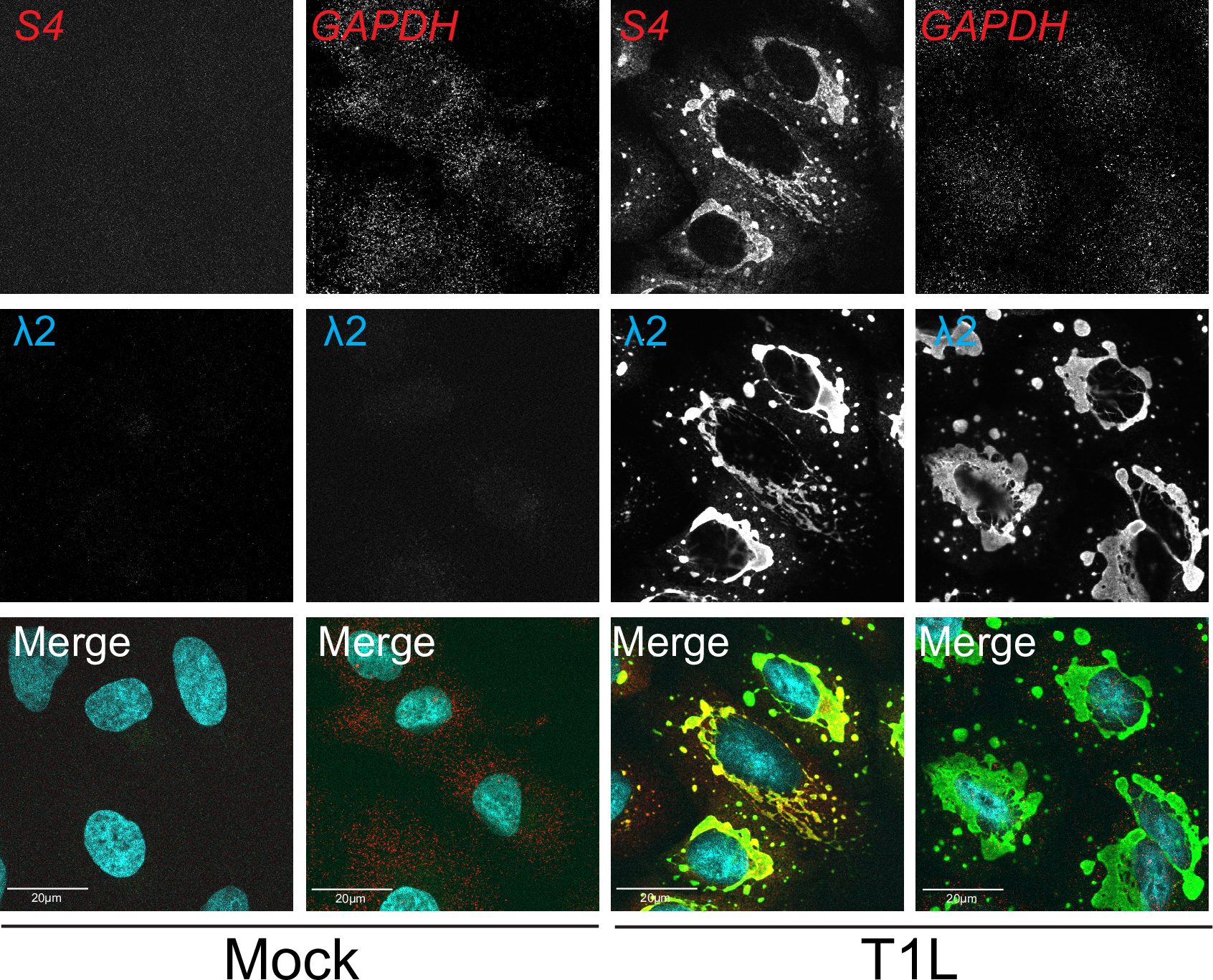

Supplement: S7 Fig — A549 cells were either mock infected or infected with T1L-WT at 100 PFU per cell. At 18 h pi, cells were fixed for immunostaining with antibodies against viral protein λ2 followed by secondary antibodies staining. Subsequently, CAL Fluor Red 610 Dye-conjugated s4 mRNA probes or Quasar 670 Dye-conjugated GAPDH mRNA probes were used to detect s4 mRNA or GAPDH mRNA, respectively. Images were collected using Olympus FLUOVIEW FV3000. (TIF) [file ppat.1009494.s011.tif]

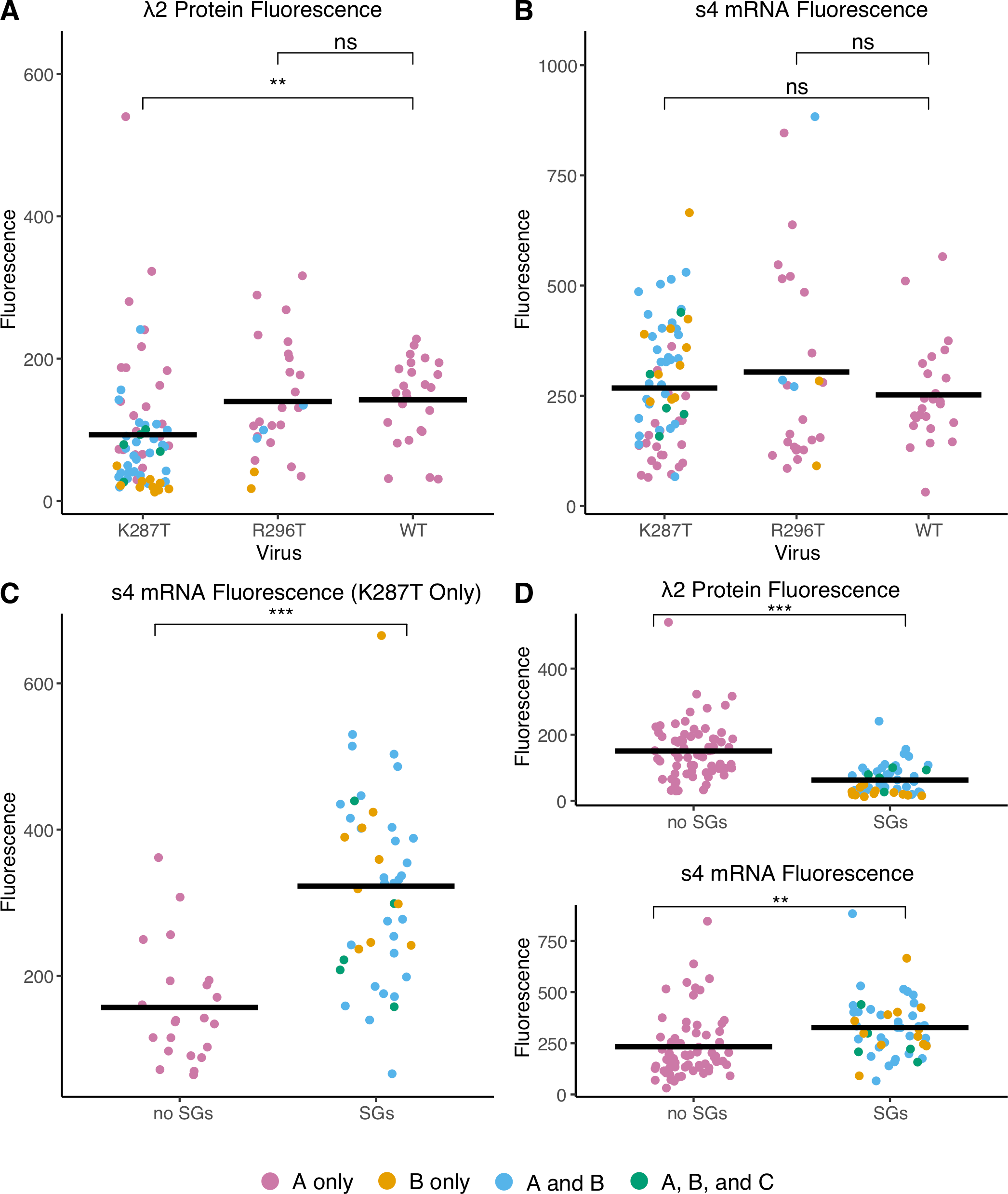

Supplement: S8 Fig — Colored circles represent individual infected cells categorized according to the distribution of s4 mRNA within each cell: A only (purple circles)—s4 mRNA co-localized within clearly defined VFs as detected by λ2 staining, SGs absent; B only–(yellow circles) s4 mRNA co-localized with TIAR in SGs; A and B–(blue circles)—a mixture of phenotypes A and B, SGs detected; A, B, and C–(green circles) a mixture of phenotypes A and B and in addition co-localization of s4 mRNA, TIAR, and λ2 within clearly defined VFs, SGs present. (A) Mean λ2 and (B) s4 mRNA fluorescence of individual A549 cells infected with WT, K287T and R296T viruses at 18 h pi. (C) Mean s4 mRNA fluorescence of K287T-infected cells grouped according to the presence or absence of SGs. (D) Mean λ2 and s4 mRNA fluorescence of all infected cells grouped according to the presence or absence of SGs. Multiple comparison, non-paired t tests were used to analyze differences, comparing with WT or no SGs, (ns = not significant; **, P < 0.01, ***, P < 0.001. (TIF) [file ppat.1009494.s012.tif]

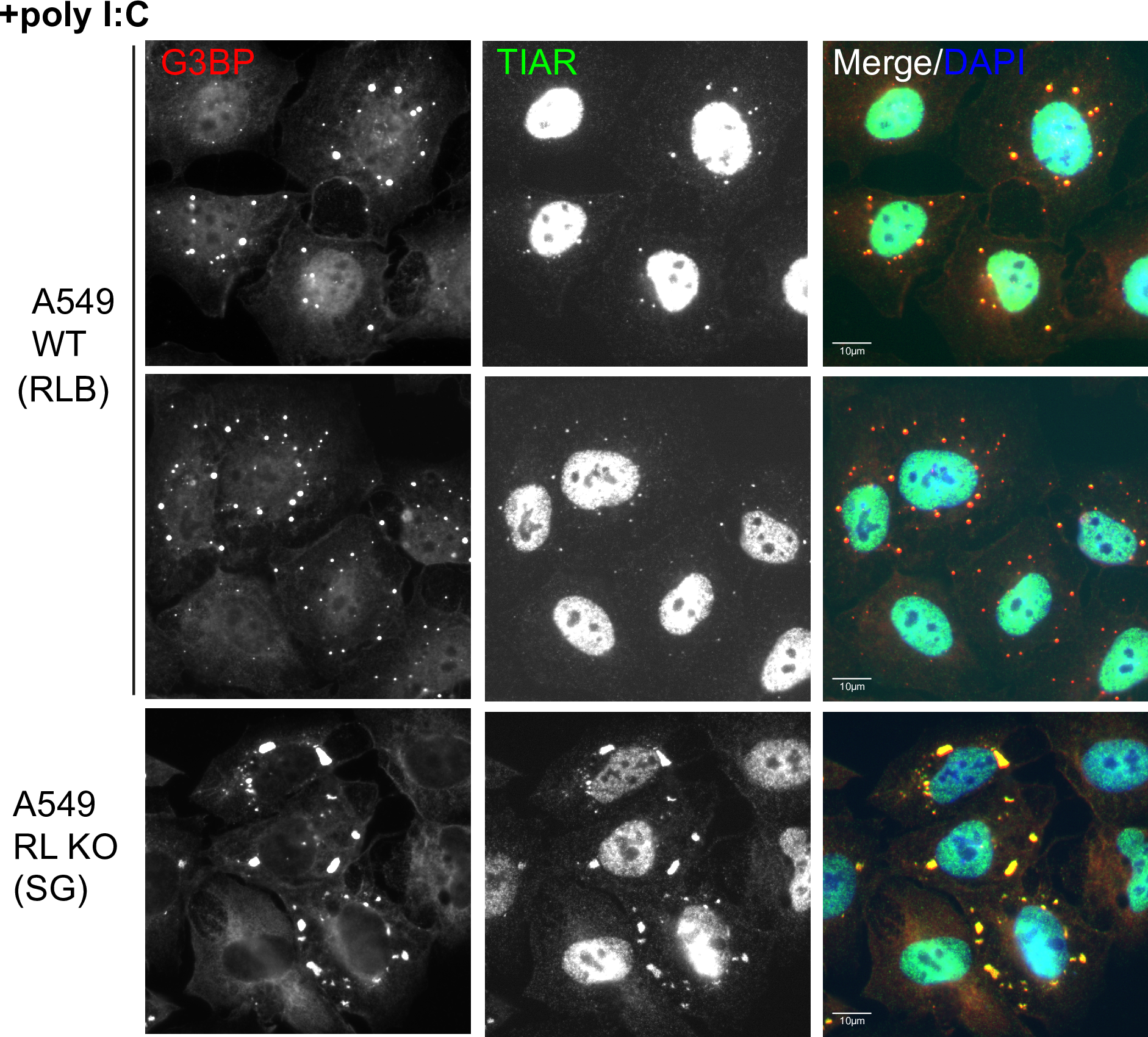

Supplement: S9 Fig — A549 cells or A549 RNase KO cells were transfected with poly I:C at 500 ng/ml using Lipofectamine 2000 following the manufacturer’s instructions. At 8 h post-transfection, cells were fixed and immunostained for G3BP and TIAR. Nuclei were counterstained with DAPI. (TIF) [file ppat.1009494.s013.tif]

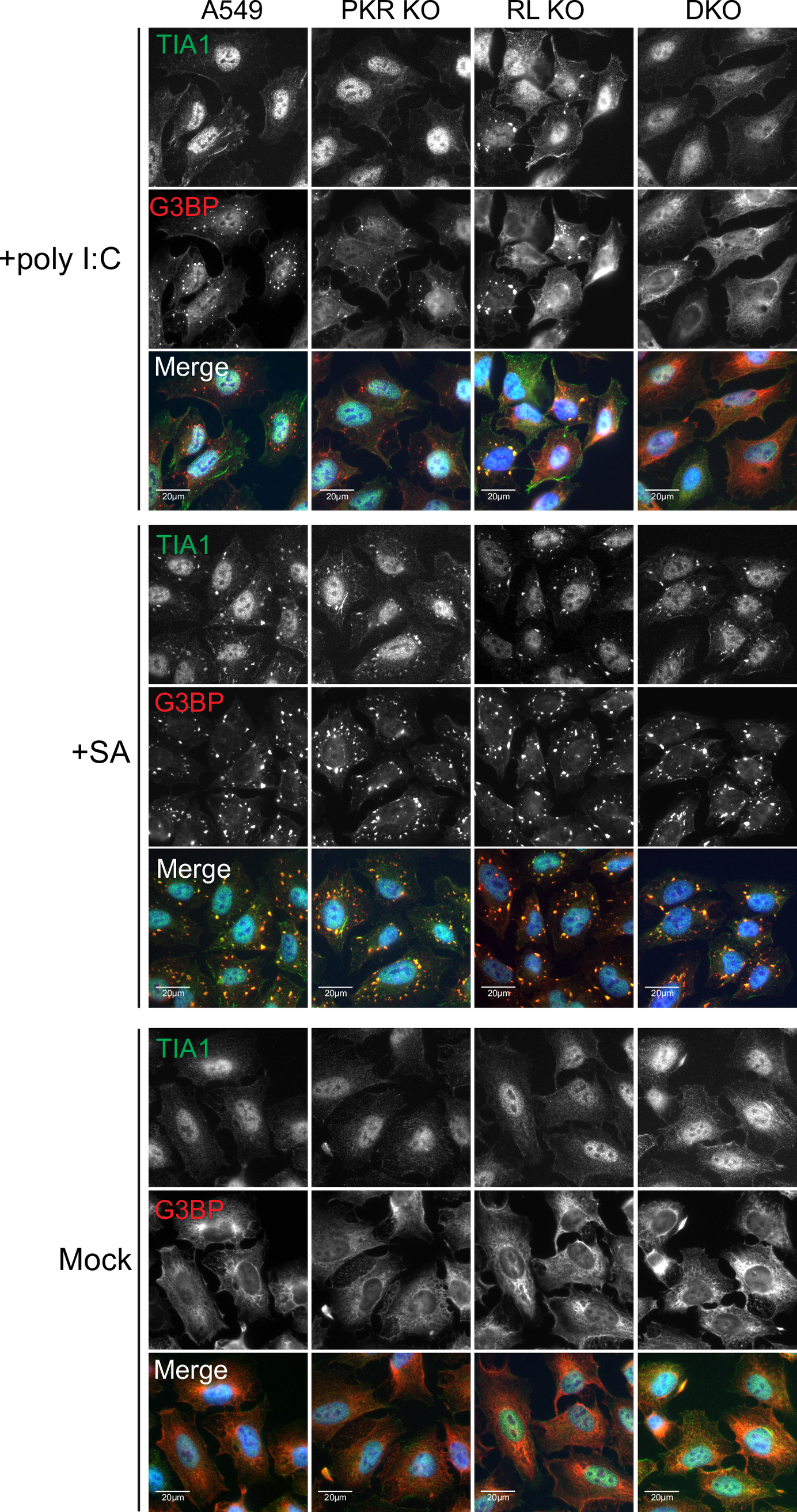

Supplement: S10 Fig — Cells as indicated were transfected with 500 ng/ml poly I:C and then incubated for 8 h (upper) or treated with 0.5 mM sodium arsenite for 1 h (middle) or left untreated (bottom). After transfection with poly I:C or treatment with SA, the cells were fixed and immunostained for TIA1 and G3BP. Nuclei were counterstained with DAPI. (TIF) [file ppat.1009494.s014.tif]

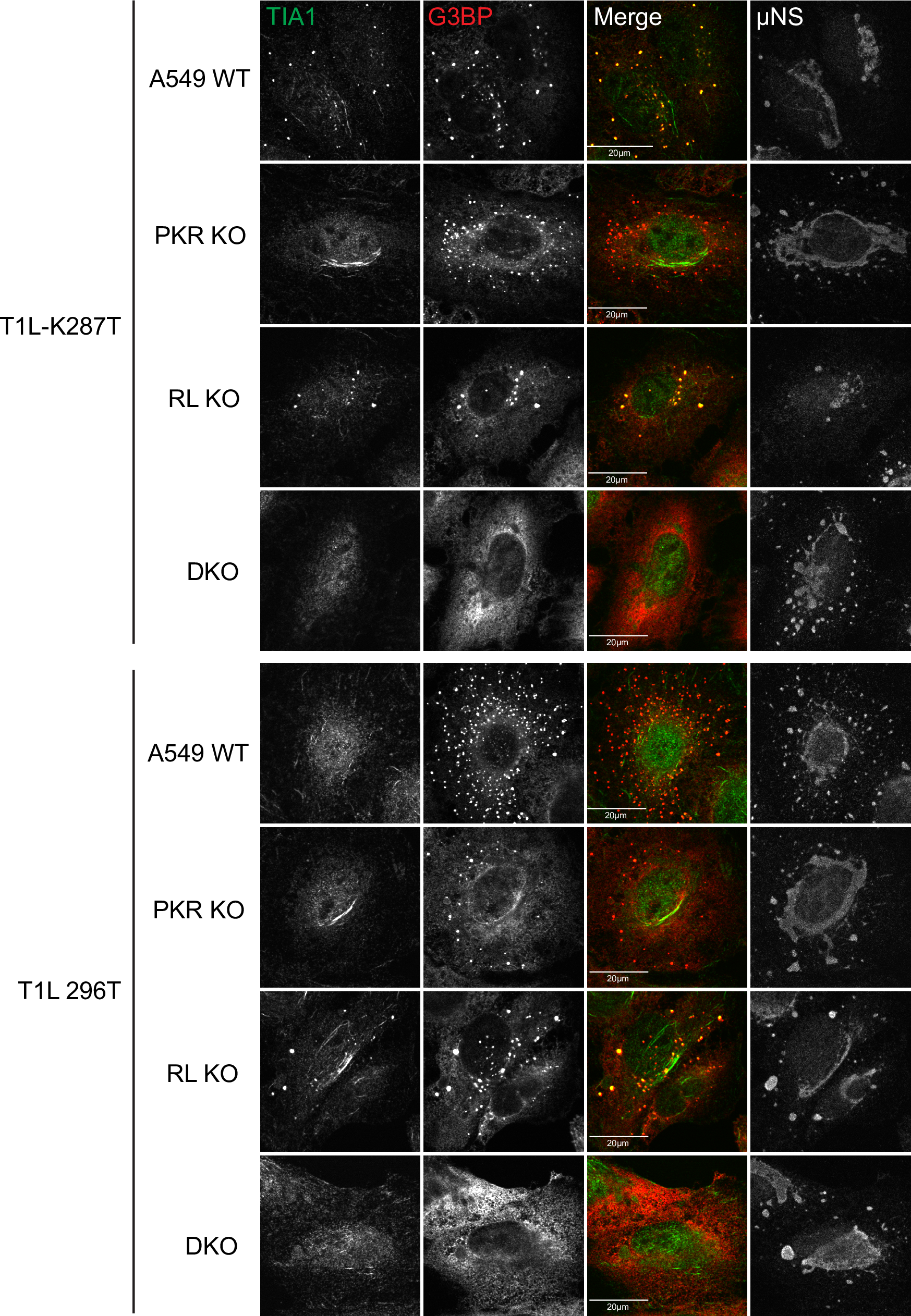

Supplement: S11 Fig — Cells as indicated were infected with either T1L-K287T or T1L-R296T virus at 100 PFU per cell. At 18 h pi, cells were fixed and immunostained for TIA1, G3BP and μNS. Merged image is showing colocalization of TIA1 and G3BP1. Images were collected using Olympus FLUOVIEW FV3000. (TIF) [file ppat.1009494.s015.tif]

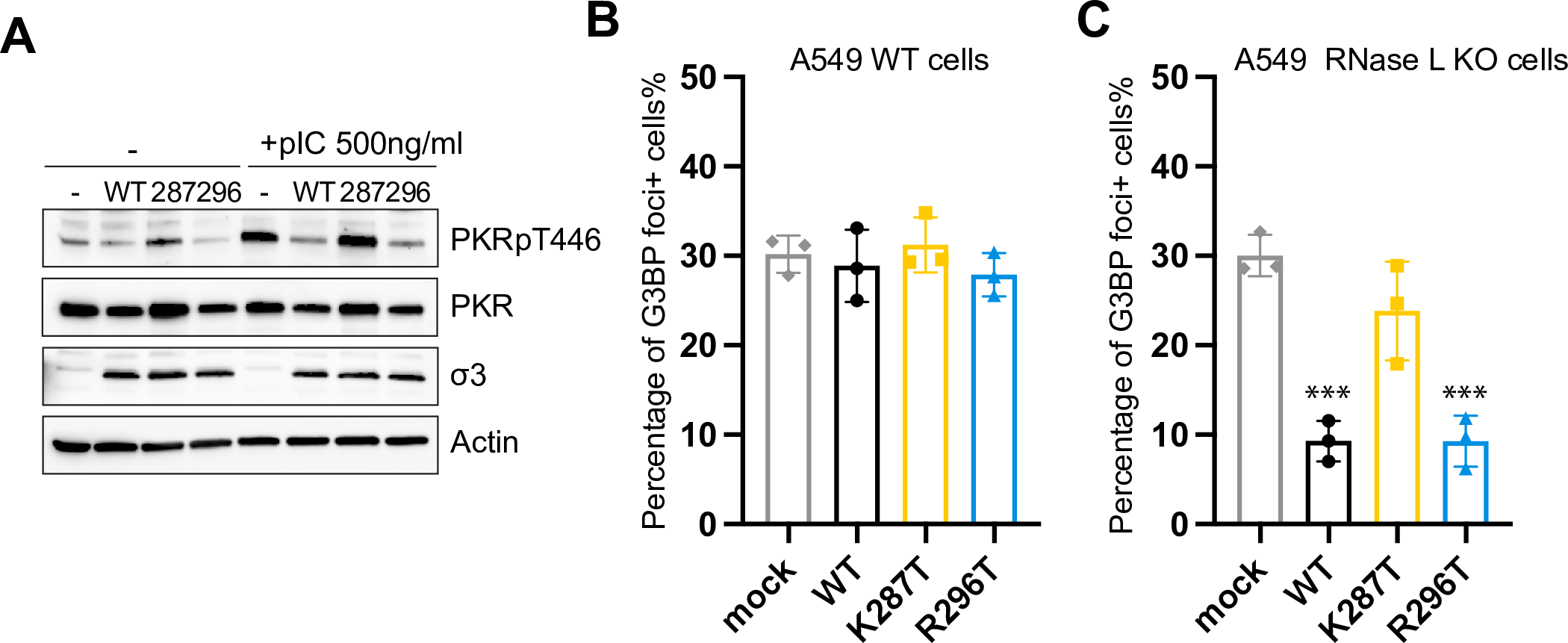

Supplement: S12 Fig — (A) Ectopic expression of WT σ3 or the R296T mutant, but not the K287T mutant prevents phosphorylation of PKR. σ3 protein expression was induced by treatment with doxycycline (1 μg/ml) for 24 h. Cells were then transfected with 500 ng/ml Poly I:C. At 6 h post-transfection, lysates were collected for analysis of the phosphorylation status of PKR by immunoblot. (B) Ectopic expression of WT or mutant σ3 in WT A549 cells does not prevent poly I:C-induced RLB formation. Cells were induced and treated as before, but at 6 h after transfection cells were fixed and immunostained for G3BP and σ3. The number of σ3 positive cells containing > 3 foci of G3BP-positive granules were counted (> 200 cells were counted per biological replicate). (C) Ectopic expression of WT or R296T mutant σ3, but not K287T prevents formation of SGs in RNase L KO cells treated with poly I:C. Data shown represent the mean ± s.d. of three independent experiments. Multiple comparison, non-paired t tests were used to analyze differences compared to mock. (ns = not significant; ***, P < 0.001). (TIF) [file ppat.1009494.s016.tif]

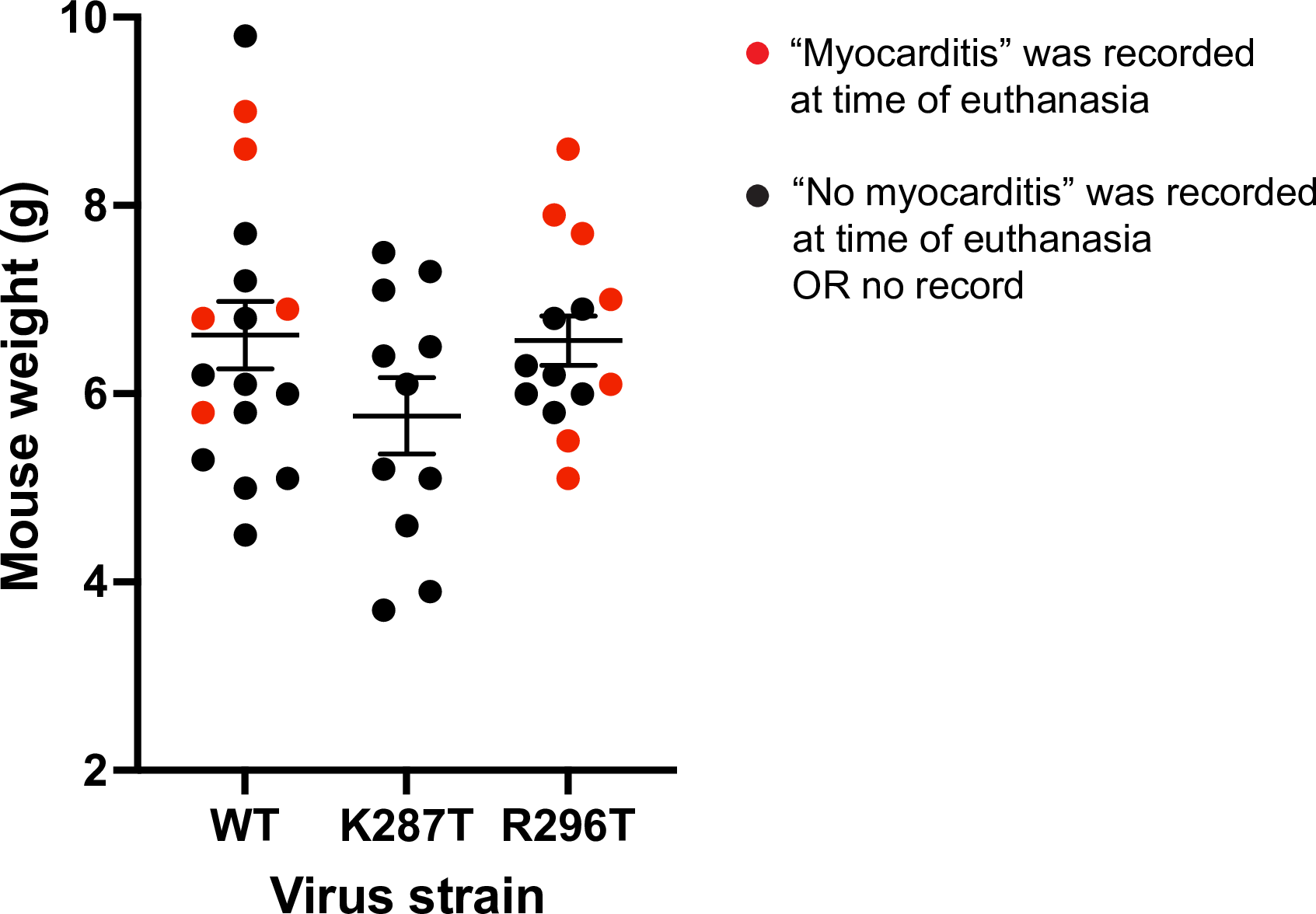

Supplement: S13 Fig — C57BL/6J 3-4-day-old mice were inoculated perorally with 107 PFU of WT, K287T, or R296T virus. Mice were weighed and euthanized 8 d pi for either RNA/histology or viral titer experiments. Each symbol indicates the weight in grams of one mouse. Mean and SEM are shown. Groups were not statistically different by ANOVA (*, P > 0.05). (TIF) [file ppat.1009494.s017.tif]
