# Supplementary material for: The multi-functional reovirus σ3 protein is a virulence factor that suppresses stress granule formation and is associated with myocardial injury
Source: PLoS Pathog. 2021 Jul 8;17(7):e1009494. doi: 10.1371/journal.ppat.1009494 (PMC8291629; doi:10.1371/journal.ppat.1009494)
Supplement: Attachment — Submitted filename: PPATHOGENS-D-21-00634 Response to Reviewers Final.docx [file ppat.1009494.s018.docx]

**PPATHOGENS-D-21-00634 (The multi-functional reovirus σ3 protein is a virulence factor that suppresses stress granule formation to allow viral replication and myocardial injury) – Responses to reviewers:**

Dear Editors,

We appreciate the efforts of the reviewers and editors in providing a prompt and thoughtful review. We have responded to all of the reviewers’ questions and suggestions as described below. In addressing these critiques, we have made changes to the text and added five new supplementary figures as well as new data to two of the original figures and one of the original supplementary figures. The reviewers’ comments are numbered and highlighted in *italics*; our responses are in regular text. We have indicated the page and line number in the updated manuscript, where changes to the text of the manuscript have been made in response to a reviewer’s suggestion/critique. We also have submitted a clean version of the revised manuscript and a copy with changes highlighted in yellow. We look forward to a favorable response to our revised manuscript.

**REVIEWER #1.**

**Major concerns:**

*1. As outlined above, testing binding to viral dsRNA may be informative as to more subtle differences in RNA binding between the mutants.*

Response: To address this comment, we purified and biotinylated viral dsRNA from purified T1L virions and used the biotinylated viral dsRNA in pulldown assays with WT σ3 and the mutant forms of K287T and R296T σ3 (see new supplementary Figure S1). As we found for poly I:C pulldowns (Fig. 1), biotinylated viral dsRNA was efficiently precipitated by WT σ3 but not by the K287T or R296T mutant forms of σ3. These new data are described in the results section lines 181 to 185. In addition, a new section has been added to the Materials & Methods section describing purification of reovirus virions and biotinylation of dsRNA (lines 824-833).

*2. Although not necessary to justify the main conclusions of the manuscript, inclusion of competition assays for dsRNA binding between* σ*3 and PKR, or testing for direct interactions between* σ*3 and PKR, may also be highly informative to the mechanism for* σ*3 inhibition of PKR.*

Response: We agree with the reviewer that these assays may be highly informative, and we have initiated these experiments. We found that WT σ3 and the R296T mutant, but not the K287T mutant, co-immunoprecipitate with PKR in the absence of exogenous dsRNA. This finding suggests that a direct interaction of σ3 with PKR prevents PKR phosphorylation. However, the story appears to be more complicated. In tandem affinity purification experiments using σ3 as bait, we identified ILF3 (NF90) as a protein that interacts with σ3. ILF3 has been implicated in the regulation of stress granule formation and PKR activation (1–3). Both the WT and R296T mutant co-immunoprecipitate with ILF3, but the K287T mutant does not, indicating that the dsRNA-binding capacity of σ3 is not required for these protein-protein interactions. Influenza NS1 antagonizes PKR by interacting with NF90 and PKR simultaneously, preventing the PKR-activating function of NF90 (4). We hypothesize that a similar mechanism exists for σ3. We expect that our ongoing experiments will unravel the functional and mechanistic significance of these interactions, but we think these studies are beyond the scope of this manuscript.

*3. An assessment of the particle:PFU ratio, as well other assessments of particle stability (beyond trypsin sensitivity) would help to exclude other potential confounders to the interpretation of the data.*

Response: Reported particle-to-PFU ratios for WT reoviruses vary considerably between preparations (between 100 to 1,000) (5, 6). The particle-to-PFU ratios for purified WT T1L, K287T, and R296T viruses used in this study were 184, 430, and 158, respectively. All are within the range reported for purified virions. We have added text to the Results section (lines 258-259) to state these findings.

*4. Fig. 4: Given the decreased viral protein production of the T1L-K287T mutant, use of immunofluorescence for* λ*2 is not the best marker for reovirus-infected cells (some of the cells noted as “infected” appear to have very little to no* λ*2 staining. The authors use smFISH in subsequent figures, which could be a better proxy to label infected cells (although, given the high MOI used, restricting the analysis of granules only to infected cells may not be necessary to show an overall increase in granule-containing cells in the experiment).*

Response: All cells infected with the K287T mutant expressed low levels of λ2 and were clearly distinguishable from uninfected controls. We chose to use the 7F4 monoclonal antibody against λ2 specifically to detect infected cells where low levels of protein are expressed, as this antibody shows very low background staining in uninfected cells allowing low levels of protein signal to be reliably detected. This is difficult to show in immunofluorescence images but is clearly visible by microscopy. We have digitally enhanced the signal in the K287T-infected cells panel in Figure 4 to better show which cells were infected and made note of this in the figure legend. Only granules in cells that were clearly infected were counted.

*5. The authors do not mention any measures of the overall effect of infection on disease status in the animals. Did any of the animals succumb to myocarditis? Did they exhibit weight loss or other pathologic signs or sequelae? A bit more characterization of the disease manifestations elicited by the mutant viruses would help in interpreting the overall pathologic impact. Along these lines, based on the figures, it appears that (perhaps with a single exception) the R296T virus may have caused even more significant myocarditis than T1L, which would be a very intriguing phenotype. More exploration of this could prove very interesting – would complete loss of dsRNA binding attenuate, but a partial effect (or retaining PKR inhibition in the absence of dsRNA sequestration) lead to a more severe disease phenotype?*

Response: Because of the early time points chosen for these studies (4- and 8-days post-infection), none of the animals succumbed to myocarditis or other sequelae associated with T1L infection in newborn mice. Animal weights were recorded on each day and showed no significant changes between groups over the course of the infection, but the weights trended higher in WT- and R296T-inoculated mice than in K287T-inoculated mice at day 8 post-infection (see lines 470-474) (data shown in Fig. S13). Excess weight would be consistent with fluid retention associated with heart failure in myocarditis. We are currently exploring whether R296T virus promotes increased incidence or severity of myocarditis relative to WT virus. However, we have found no evidence of partial dsRNA binding for the R296T mutant. Both the K287T and R296T have no apparent dsRNA binding capacity in experiments using either poly I:C or viral dsRNA.

**REVIEWER #2.**

*1. The fact that viral mRNA levels are increased for the mutant virus K287T although viral replication and protein synthesis are attenuated is very intriguing. The authors propose that reduced viral translation is likely due to the host translational repression and mention line 257 that this mutant induced increased p-PKR and p-eIF2α levels. However, this claim is not supported by the Western blot analysis shown in Figure 3D. While p-PKR levels are indeed clearly increased, an increase in p-eIF2α levels is not detectable. Based on this, the conclusion appears to be too preliminary. Is cellular translation repressed? To support this point, the authors should perform a ribopuromycin assay, which allows for the measurement of puromycin incorporation and thereby de novo protein synthesis, either by fluorescence microscopy or by Western blot analyses. In addition, the number of repeats and a quantification of p-PKR and p-eIF2α levels should be provided, as done for Figure 3C.*

Response: We agree with the reviewer that the original blot shown in Fig. 3D had a relatively weak band for p-eIF2α for the mutant virus K287T. However, there was a band present. We have replaced the blot with a better representation showing that the levels of p-eIF2α are clearly increased in the K287T mutant relative to the WT and R296T mutant in Fig. 3D. In addition, Fig. 6C also definitively shows phosphorylation of eIF2α in the K287T-infected WT and RNAse-null A549 cells. The requested quantification of p-PKR and p-eIF2α for three independent immunoblot experiments is now included in Figure 3F. Additionally, as suggested by the reviewer, we conducted ribo-puromycylation (SUnSET) assays in A549 cells to compare the incorporation of puromycin in WT-, K287T-, and R296T-infected cells. The findings indicate that global cellular translation is significantly repressed in K287T-infected cells relative to mock-, WT-, and R296T-infected cells, consistent with the presence of phosphorylated eIF2α. We have added text to the Results section describing these additional findings (lines 286-294) and show the data in supplementary Fig. S6.

*2. To determine the potential SG-suppressive function of σ3 mutant, the authors analyzed the number of TIAR-positive cells in response to wild-type and mutant virus infection (Figure 4B). Some statements are unclear in this section to this reviewer. The authors mention line 271 that in the case of wild-type virus, SG formation peaks at about 6 h post-infection (hpi) and is repressed thereafter (24 hpi). In work by the Miller lab they refer to, infection induces SGs in nearly 40% of infected cells between 4 and 6 hpi. This number decreases drastically already at 12 hpi. In the histogram shown in Figure 4B, the number of SG-positive cells induced by infection with the wild-type virus remains low, less than 5%, and mostly unchanged between 6 and 18 hpi. Is this discrepancy due to the fact that SG were analyzed in all cells and not only infected cells? Is it due to the use of a different cell type and therefore different kinetics? From the result shown in 4B, we can only conclude that this wild-type strain induces little or no SGs.*

Response: The reviewer is correct that the frequency of SG-positive cells following WT T1L infection is low (3-5%) relative to that reported by the Miller lab (7). There are two reasons for this discrepancy. First, the rate of 40% SG-positive cells reported by the Miller lab was for infections using infectious subvirion particles (ISVPs) at an MOI of 1000 PFU/cell. Induction of detectable SGs by T1L virions is hypothesized to be lower due to delayed entry kinetics relative to other strains. However, as we demonstrate, T1L does induce detectable SGs in a small percentage of cells. Second, we used A549 cells in our experiments, whereas the paper from the Miller lab used DU-145 prostate cancer cells, HeLa cells, mouse embryonic fibroblasts, and CV-1 monkey kidney cells. Qin et al. noted that they did not observe any SGs in the cells they used when infecting with T1L virions and they hypothesized that this result was due to delayed virus entry of the T1L strain. They repeated their experiments using ISVPs, which can penetrate directly from the plasma membrane, and found that all strains induced SGs to ~ equivalent levels in a dose-dependent manner (7). We have conducted similar experiments and the results are shown in the figure below but are not included in the paper. We have revised the text to make this point clear (lines 303-307). Please also see Discussion lines 669-673.

G3BP-positive stress granules in HeLa and A549 cells infected with wildtype T1L ISVPs at 4 h pi. Cells were infected at MOIs of 100 or 1000 PFU per cell. Cells were fixed and immunostained for G3BP and reovirus cores at 4 h pi. A minimum of 200 cells were counted per condition. The percent of SG-positive infected cells is shown.

*3. Related to the previous point. Although this reviewer agrees that more SG-positive cells are detected upon infection with the mutant virus K287T at a later time point, it cannot rule out whether this virus simply induces more SGs because of increased p-eIF2α levels. The direct evidence showing that the mutant virus suppresses SGs after their initial formation, as concluded in line 283 is missing. This result could be easily addressed by performing an experiment in which cells infected with both wild-type and mutant viruses are treated with arsenite.*

Response: We agree with the reviewer that the K287T mutant is likely inducing more SGs because it induces increased levels of p-eIF2α. However, it is also clear that this mutant cannot prevent or suppress SG formation. Co-expression of the viral nonstructural proteins μNS and σNS is reported to suppress SGs independently of increased levels of p-eIF2α (8). However, cells infected with the K287T mutant form SGs, and in the face of ongoing phosphorylation of eIF2α, μNS and σNS cannot suppress SG formation in such cells. To alleviate the reviewer’s concern, we conducted the suggested arsenite experiments. Following treatment of K287T-infected cells at 18 hpi with arsenite, we found no difference in the level of p-eIF2α or in SG-positive cells between untreated and arsenite-treated cells. These results are difficult to interpret, as ~55% of K287T-infected cells have SGs and high levels of p-eIF2α prior to treatment with arsenite. One could infer that μNS and σNS are acting to suppress arsenite-induced SGs as has been previously reported. However, we were unable to show an increase in eIF2α phosphorylation in response to arsenite in K287T-infected cells, thus making it difficult to know whether the cells were unable to respond or, alternatively, that the level of phosphorylation of eIF2α had reached a maximum. Qin et al. interpreted the lack of induction of SGs by sodium arsenite in infected cells as an indicator of suppression of SGs, despite the presence of increased levels of p-eIF2α (9). In the case of the K287T mutant, the levels of p-eIF2α were high prior to treatment with sodium arsenite.

We recognize that the word ‘suppress’ versus ‘prevent’ may cause some confusion. Therefore, we modified the text to clarify this point. In addition, we added new data demonstrating that ectopic expression of WT σ3 and the R296T mutant, but not the K287T mutant, prevents PKR phosphorylation in response to poly I:C in WT and RNase L KO A549 cells. In addition, the formation of poly I:C-induced SGs in RNase L KO cells was prevented by ectopic expression of WT and R296T σ3, but not by K287T (see Results, lines 437-456). However, σ3 expression did not prevent formation of poly I:C-induced RLBs in WT A549 cells (Fig. S12) nor did it prevent arsenite-induced SGs (data not shown).

*4. The authors used immunofluorescence analysis combined with FISH to visualize differences in S4 mRNA abundance in viral factories (VFs) or SGs for the different viruses, for example, less S4 mRNA is found in VFs for the mutant virus K287T. While this analysis can be very informative and the images are of very good quality, it lacks quantifications to support the different claims and highlight the observed differences. We can hardly conclude on the basis of the provided representative still images. Quantification would also avoid using terms such as "often co-localized" or "mostly co-localized" that are only poorly indicative. In addition, the number of cells analyzed in Figure 5 should be indicated.*

Response: We agree with the reviewer and have provided a quantitative analysis of the immunofluorescent FISH data (Fig. S8). We have also modified the text to remove co-localization comparisons, as they are hard to interpret when infected cells show a mixture of phenotypes. Instead, we classified the infected cells into four phenotypes: (A) S4 mRNA co-localizes within clearly defined viral factories as detected by λ2 staining; (B) S4 mRNA co-localizes with TIAR in SGs; (C) S4 mRNA, TIAR, and λ2 co-localize within clearly defined viral factories; and (D) a mixture of phenotypes A and B, where TIAR does not localize to clearly defined viral factories. We analyzed the mean fluorescence per cell of S4 mRNA and λ2. We found significant differences in the mean levels of λ2 protein per cell between K287T- and WT-infected cells. However, unlike for protein levels (Fig. 3A), we did not find a significant difference between K287T-, R296T-, and WT-infected cells in the mean fluorescence levels of S4 mRNA in the total population of infected cells analyzed. However, when the K287T data were re-analyzed on the basis of cell phenotype, we found that cells containing TIAR-positive SGs had significantly higher S4 mRNA mean levels per cell than cells that lacked SGs. When these data were analyzed for all three viruses (WT, K287T, and R296T), we found that cells with TIAR-positive SGs had significantly higher S4 mRNA levels per cell and lower λ2 protein levels per cell relative to infected cells that lacked SGs. These new results are described lines 335-354.

*5. The authors show that as described previously, TIA1 is a SG-specific marker that is not detected in RNase L Bodies (RLBs). How much does the quantification shown in Figure 6A differs when analysis TIA1-specific SGs? This additional analysis would strengthen the difference between the K287T and the R296T mutant viruses.*

Response: We do not think additional analysis of TIA1 will clarify the data shown in Fig. 6A. In the absence of a specific marker for RLBs, it is unfortunately not possible to accurately differentiate RLBs from SGs in WT cells. Our understanding of the reviewer’s question is that by using TIA1 we would only count SGs, whereas our current data using TIAR as a marker counts both SGs and RLBs. By definition, RLBs cannot form in cells that lack RNase L. The data shown for RNase L-null cells (green bars) demonstrate a significant difference in the induction of SGs by the K287T mutant (~ 60% of infected cells) compared with the R296T mutant (~ 10% of infected cells) (Fig. 6A). In contrast, the percentages of K287T- and R296T-infected cells containing TIAR+ granules do not significantly differ in PKR-null cells (pink bars) in which PKR-dependent SGs cannot form. Our interpretation of these findings is that in the absence of PKR, both mutants induce TIAR+ granules in ~ 10% of infected cells, which we demonstrate are RLBs due to the complete absence of TIA1 signal (Fig. S10). The granules formed in RNase L-null cells infected with K287T and R296T contain TIA1 and G3BP, indicating that these are SGs.

*6. While the mouse experiment is interesting and provides in vivo information about the mutant virus, it is not clear what this piece of data along with the transcriptomic analysis actually contributes to the SG aspect. There is no evidence of a direct link to PKR in vivo. As the authors themselves mention in line 404, one can only speculate that the reduced myocarditis is due to the initial formation of SGs by the mutant virus K287T. This could alternately be a result of the reduced level of replication. For this reason, the title should be reconsidered.*

Response: We agree with the reviewer and have modified the title of the paper to better reflect our findings. The new title: “The multi-functional reovirus σ3 protein is a virulence factor that suppresses stress granule formation and is associated with myocardial injury”, does not state a direct relationship between the two phenotypes of SG formation and myocardial injury.

**REVIEWER #3.**

*1. The authors initially screen sigma-3 RNA binding by examining the interaction with poly I:C, a mimic of double-stranded RNA. It is notable that both K287T and R296T are deficient in binding poly I:C, yet only K287T shows a phenotype.

a. Is there a difference in the ability of the K287T and R296T mutants to interact in vitro with reovirus double-stranded RNA or in cells with viral mRNA, negative-sense RNA and double-stranded RNA during infection? Showing such a difference would provide additional support towards the proposed model.*

Response: Please see our response to question 1 from reviewer #1.

*b. Lines 118-120: the authors note that the amount of free sigma-3 (or not in complex with mu-1) might affect translational shutoff. Is there a difference in the abundance of free sigma-3 between the K287T and R296T mutants that might support this hypothesis and/or the author’s model?*

Response: We did not specifically assess the levels of free σ3 in infected cells for the WT, K287T or R296T viruses. However, it is clear that there is less σ3 expressed in K287T-infected cells (Fig. 3C). The T1L strain has been reported to induce moderate cellular translational shutoff at high multiplicity (MOI = 80 PFU/cell) in L cells (10). To determine the level of translational shutoff, we conducted puromycin labeling experiments in A549 cells that were infected with WT, K287T, or R296T at an MOI of 100 PFU/cell (Fig. S6). We detected no differences in the levels of puromycin incorporation in mock-, WT-, or R296T-infected cells when the total amount of puromycin incorporated per gel lane was quantified. However, consistent with previous findings (10), levels of host proteins greater than 100 kDa in size were decreased relative to mock in WT- and R296T-infected cells. In contrast, the K287T mutant was associated with significantly decreased levels of overall protein synthesis, including viral protein synthesis, consistent with our findings that K287T-infection induces high levels of phosphorylated PKR, phosphorylated eIF2α, and increased numbers of SGs. Although it is possible that there is a difference in the levels of free σ3, we think that unlikely for the following reasons: (i) the capacity of the mutant σ3 proteins to assemble with μ1 did not appear to differ when μ1 and σ3 were co-expressed (Fig. S2B); (ii) the sites of the R296T and K287T mutations in σ3 are not within σ3-μ1 or σ3-σ3 dimer interaction interfaces; and (iii) we found that ectopic expression of WT or R296T σ3 alone was sufficient to prevent poly I:C-induced PKR phosphorylation, whereas the K287T mutant was unable to prevent PKR phosphorylation (Fig. S12). These data indicate that, independent of any potential difference in the levels of free σ3 within infected cells, the K287T and R296T mutants differ in the capacity to inhibit PKR phosphorylation. Taken together, the preponderance of evidence fits the model presented in the manuscript and does not support a model whereby the phenotypes we describe can be ascribed to differences in levels of free σ3 within infected cells.

*2. While the authors clearly show a difference between the K287T and R296T, it would be good to include in the discussion why R296T (even though the mutant has a clear defect in binding Poly I:C) does not show the same phenotype as K287T. While this is likely speculation, or the basis of a future manuscript, this would provide additional context to the paper.*

Response: We have preliminary findings indicating that WT σ3 and the R296T mutant can interact with PKR, whereas the K287T mutant cannot. In addition, we have identified several host proteins, including ILF3, that can interact with WT σ3 and R296T, but cannot interact with K287T. The mechanism by which σ3 inhibits PKR will be the focus of a future study. In response to the reviewer’s comment, we have added some text to the Discussion speculating about the possible reasons for the difference in phenotypes between the mutants despite their shared incapacity to bind poly I:C or viral dsRNA (lines 561 to 574).

**Minor Issues: Editorial and Data Presentation Modifications.**

**Reviewer 1.**

*1. Fig. 3: Panel B is referred to first in the results text, followed by panel C, then panel A. Either relabel the figure, or reorder the results paragraph.*

Response: Thank you for pointing this out. We have re-ordered the Figure 3 panels to reflect their order of first appearance in the text.

*2. There are some tantalizing differences in RNA synthesis and protein synthesis between the mutants and wild type T1L, with only 3 replicates. Is the N high enough to really statistically power the results? Additional replicates may help to allow the authors to discuss these more subtle difference, which may, nevertheless, impact virus replication and pathogenesis.*

Response: We agree with the reviewer’s comment. However, we would need to redesign and repeat the experiment to test the hypothesis that the WT and R296T mutant differ. We estimate that would require six independent biological replicates. To add replicates post-hoc would not be appropriate and likely not generate statistically valid data. The current data show a statistically significant difference between WT and the K287T mutant. We plan in future experiments to independently examine the phenotype of the R296T mutant, which can suppress PKR activation but that we suspect has other phenotypic differences from WT.

*3. Line 227/285/etc.: The authors repeatedly state that reovirus induces SG early in infected cells (citing refs. 27 and 28). These experiments were only done using T3D, and in different cell types (e.g., HeLa), and their own data suggests that WT T1L does not induce SGs at an early time in A549 cells, rather that T1L-K287T fails to continue to suppress SG formation at later times at infection. This language should be clarified, as there appear to be serotype-/cell-type specific effects that may not warrant the broad claim as a “universal” reovirus phenotype.*

Response: Qin et al. used the T1L, T2J, and T3D-Cashdollar reovirus strains and found that all three induced SGs early after infection to varying degrees. They tested these viral strains in HeLa, CV-1, MEFs, and DU-145 cells. T1L virions induced few or no SGs, which Qin et al. attributed to a delay in virus entry, as T1L, T2J, and T3D-Cashdollar ISVPs induced similar levels of SGs at early times in all cells tested (7). The different findings made using different cells relates to strain-specific differences in virus attachment and entry that are unrelated to the capacity of each strain to induce SGs. We have added clarification to the text as requested (lines 303-307).

*4. Line 295: the authors make a comparative claim, that the “level of colocalization was less than that in WT-infected cells.” There is no quantitative data to support this claim, so it should be amended, or quantification should be provided.*

Response: We thank the reviewer for pointing this out. We have amended the text to avoid claims based on ‘levels’ of colocalization.

*5. Line 302: The authors indicate that the R296T mutant still sequesters viral RNA in SGs, and that “the resultant sequestration impairs viral replication,” yet R296 does not exhibit a replication defect. Please clarify.*

Response: We thank the reviewer for pointing this out. We have removed the reference to viral replication (see lines 332-334).

*6. Figure 7: There appear to be some issues with the asterisks indicating statistical differences (at least in the PDF as viewed on screen, versus printing).*

Response: The asterisks have been corrected.

*7. Given the (perhaps not quite statistically significant) enhancement of myocarditis by the R296T mutant, it would be interesting to have some more data on the 41 genes solely induced by this mutant (Fig. 9) – do these give any indication as to a potential phenotype? Overall, the RNA-seq data is underexplored, as there may be some useful information there; addition of a second time point (at perhaps day 8), could really shed some light on to the mechanism for the less severe phenotype induced by T1L-K287T (and potentially the more severe phenotype induced by R296T).*

Response: The differentially expressed genes are presented in a new supplementary table (Table S3). The RNA-seq data did not provide obvious candidates that could explain the phenotypic differences between the R296T and K287T mutants. To further investigate differences between the viruses, we conducted spatial transcriptomic analysis combined with scRNA-seq to identify cell-specific differences within infected tissues. The analyses of these data in the heart indicate that there are differences in endothelial cell and T cell gene expression in the heart and spleen that provide insight into the differential host response to the viral mutants. These findings will be reported in a separate manuscript, as this is an exceptionally rich data set that provides extensive additional information on the host response to reovirus-induced myocarditis.

*8. Line 475: The authors interpret the increased viral RNA levels in the T1L-K287T to the sequestration of the RNA, making it less prone to degradation/turn-over, but there are other possible interpretations. Could the dsRNA-binding capacity of σ3 alter the overall transcription of the viral RNAs, perhaps as a means of preventing too much RNA synthesis, leading to a more robust innate immune response? A little more speculation here might be warranted.*

Response: We have added additional possible explanations for the increase in viral mRNA seen in K287T-infected cells. The hypothesis that the dsRNA-binding capacity of σ3 regulates transcription seems unlikely to us, as only cells infected with the K287T mutant had significantly increased levels of viral mRNA relative to WT-infected cells. In addition, σ3 itself does not associate with viral core particles, so it seems unlikely that changes in the dsRNA-binding capacity of σ3 per se would directly alter viral transcription. However, it is possible that the dsRNA-binding capacity of σ3 is involved in assortment of viral mRNAs for packaging into viral core particles. Antczak and Joklik reported finding σ3 together with μNS and σNS in ssRNA-containing complexes that they hypothesized were assortment complexes (11). The dsRNA-binding capacity of σ3 could be required for its association with viral mRNA assortment complexes. Decreased assortment may prevent packaging of viral mRNA and its removal from the cytoplasm. It also is possible that the overall decrease in viral protein synthesis leads to lack of outer-capsid proteins for assembly onto viral core particles leading to prolonged core transcription in K287T-infected cells. We thank the reviewer for pointing this out, and we have added text in the discussion raising other possibilities (lines 551-558).

**Reviewer 2.**

1*. Figure S3: Quantification of Western blots and an indication about the number of repeats is missing.*

Response: The requested quantification has been added to Fig. S3, and the number of repeats (3) has been added to the figure legend.

*2. Scale bars are missing in all immunofluorescence assays.*

Response: Scale bars have been added to the revised figures.

*3. Figure 4C: please provide sill images of mock cells.*

Response: We have added an image of mock-infected cells to Fig. 4C.

*4. The number of cells analyzed in Figures S7 and S8 is missing.*

Response: The images shown are representative of the phenotype that has been reported previously (14). We were confirming the previously reported phenotype and did not quantify the percentage of SGs or RLBs for these images.

*5. A growth defect of PKR knockout cells is mentioned but not really shown. Any data to support the hypothesis that this delay is rescued in double knockout cells?*

Response: The growth defect in PKR-null cells has previously been reported and was not the focus of this paper. However, we noted that the titer produced by WT T1L in PKR-null A549 cells is significantly decreased from the titer in wildtype A549 cells. In contrast, there is no statistically significant difference between the titers of T1L in the wildtype and double-knockout A549 cells (Fig. 6B). We have included a sentence in the results section that indicates this result (lines 424-426).

**Reviewer 3.**

*The authors generated sixteen mutant proteins. Of these three mutant proteins expressed poorly, seven mutations did not affect RNA binding, and six mutations ablated RNA binding. Of those six mutants unable to bind poly I:C, why did the authors focus on K287T and R296T? The rationale for focusing on those two mutations would be good to include in the results section.*

Response: We added text to the results explaining our rationale for focusing on the K287T and R296T mutant viruses (lines 218-219). This choice was made based on the need to reduce the number of mutants analyzed for in vivo experiments and also because both mutants robustly express σ3.

**References**:

1.	Patel RC, Vestal DJ, Xu Z, Bandyopadhyay S, Guo W, Erme SM, Williams BR, Sen GC. 1999. DRBP76, a double-stranded RNA-binding nuclear protein, is phosphorylated by the interferon-induced protein kinase, PKR. J Biol Chem 274:20432–20437.

2.	Wen X, Huang X, Mok BW-Y, Chen Y, Zheng M, Lau S-Y, Wang P, Song W, Jin D-Y, Yuen K-Y, Chen H. 2014. NF90 exerts antiviral activity through regulation of PKR phosphorylation and stress granules in infected cells. J Immunol 192:3753–3764.

3.	Harashima A, Guettouche T, Barber GN. 2010. Phosphorylation of the NFAR proteins by the dsRNA-dependent protein kinase PKR constitutes a novel mechanism of translational regulation and cellular defense. Genes Dev 24:2640–2653.

4.	Li T, Li X, Zhu W, Wang H, Mei L, Wu S, Lin X, Han X. 2016. NF90 is a novel influenza A virus NS1-interacting protein that antagonizes the inhibitory role of NS1 on PKR phosphorylation. FEBS Lett 590:2797–2810.

5.	Doyle JD, Danthi P, Kendall EA, Ooms LS, Wetzel JD, Dermody TS. 2012. Molecular Determinants of Proteolytic Disassembly of the Reovirus Outer Capsid. Journal of Biological Chemistry https://doi.org/10.1074/jbc.M111.334854.

6.	Hand R, Tamm I. 1973. Reovirus: effect of noninfective viral components on cellular deoxyribonucleic acid synthesis. Journal of Virology 11:223–231.

7.	Qin Q, Hastings C, Miller CL. 2009. Mammalian Orthoreovirus Particles Induce and Are Recruited into Stress Granules at Early Times Postinfection. Journal of Virology 83:11090–11101.

8.	Choudhury P, Bussiere LD, Miller CL. 2017. Mammalian Orthoreovirus Factories Modulate Stress Granule Protein Localization by Interaction with G3BP1. J Virol 91:e01298-17.

9.	Qin Q, Carroll K, Hastings C, Miller CL. 2011. Mammalian orthoreovirus escape from host translational shutoff correlates with stress granule disruption and is independent of eIF2alpha phosphorylation and PKR. Journal of Virology 85:8798–8810.

10.	Schmechel S, Chute M, Skinner P, Anderson R, Schiff L. 1997. Preferential translation of reovirus mRNA by a sigma3-dependent mechanism. Virology 232:62–73.

11.	Antczak JB, Joklik WK. 1992. Reovirus genome segment assortment into progeny genomes studied by the use of monoclonal antibodies directed against reovirus proteins. Virology 187:760–776.

12.	Knowlton JJ, Gestaut D, Ma B, Taylor G, Seven AB, Leitner A, Wilson GJ, Shanker S, Yates NA, Prasad BVV, Aebersold R, Chiu W, Frydman J, Dermody TS. 2021. Structural and functional dissection of reovirus capsid folding and assembly by the prefoldin-TRiC/CCT chaperone network. Proc Natl Acad Sci USA 118:e2018127118.

13.	Knowlton JJ, Fernández de Castro I, Ashbrook AW, Gestaut DR, Zamora PF, Bauer JA, Forrest JC, Frydman J, Risco C, Dermody TS. 2018. The TRiC chaperonin controls reovirus replication through outer-capsid folding. Nature Microbiology 1.

14.	Burke JM, Lester ET, Tauber D, Parker R. 2020. RNase L promotes the formation of unique ribonucleoprotein granules distinct from stress granules. J Biol Chem 295:1426–1438.
